# The effect of exercise training combined with PPARγ agonist on skeletal muscle glucose uptake and insulin sensitivity in induced diabetic obese Zucker rats

**DOI:** 10.20463/jenb.2016.06.20.2.6

**Published:** 2016-06-30

**Authors:** Jae-Cheol Kim

**Affiliations:** 1 Department of Sports Science, College of Natural Science, Chonbuk National University, Jeonju Republic of Korea

**Keywords:** Exercise training, Rosiglitazone, insulin sensitivity, Glucose uptake, Skeletal muscle, Diabetic rats

## Abstract

**[Purpose]:**

Exercise training with PPARγ agonist is expected to increase glucose uptake and improve insulin sensitivity in skeletal muscle of patients with diabetes. However, its mechanisms to effect glucose uptake and insulin sensitivity in skeletal muscle are unclear.

**[Methods]:**

The mechanism of action was determined by co-treatment with PPARγ agonist- rosiglitazone and exercise training in streptozotocin induced-diabetic obese Zucker rats. Exercise training was carried out for 6 weeks (swimming, 1 h/day, 5 times/week, 5% weight/g, 32±1℃) with rosiglitazone treatment (3mg/kg/day, 6weeks).

**[Results]:**

Glucose uptake and insulin sensitivity was decreased in diabetic than normal animals. Exercise training and rosiglitazone treatment respectively increased the expression of PPAR(peroxisome proliferators-activated receptor)-α, -β/δ, -γ, PGC-1α(PPAR-γ coactivator-1α), adiponectin, GLUT-4(glucose transportor-4) and p-AMPK-α_2_(phospho-AMP activated protein kinase-α_2_) in EDL and SOL of diabetic, as compared to normal animals. Interestingly, training combined with rosiglitazone significantly increased glucose uptake and insulin sensitivity, which resulted in high expression of all molecules in diabetic than all other groups.

**[Conclusion]:**

These results indicated that exercise training combined with rosiglitazone might mediate regulation of glucose uptake and insulin sensitivity in skeletal muscle. Therefore, exercise training combined with rosiglitazone may be recommended as complementary therapies for diabetes.

## INTRODUCTION

Obesity and type II diabetes are characterized by impaired glucose uptake and reduced fatty acid oxidation. Reduced fatty acids oxidation is known to cause insulin resistance, particularly in skeletal muscle^8, 28^. Skeletal muscle is responsible for majority (>80%) of insulin-stimulated whole body glucose disposal, and hence, plays an important role in the pathogenesis of insulin resistance and diabetes^28^. The genetically obese Zucker rat (fa/fa) is a well-characterized model of insulin resistant, exhibiting a range of metabolic abnormalities including hyperinsulinemia^13^.

The thiazolidinediones (TZDs) are known peroxisome proliferators-activated receptor (PPARs) agonists and major compounds for the treatment of type 2 diabetes. Rosiglitazone, a TZDs, improves skeletal muscle glucose uptake and insulin sensitivity through regulation of target genes^13,33^. Recently, studies suggest that administration of TZD to obese animal models with insulin resistance and diabetes patients can lead to the improvement of glucose uptake and insulin sensitivity associated with increasing glucose transportor-4 (GLUT-4), AMP activated protein kinase (AMPK), and PPAR-γ coactivator-1α (PGC-1)^13, 20^.

PPARs is a family of nuclear hormone receptors that include PPAR-α, -β/δ, and -γ subtypes of. It is activated by fatty acid metabolism and regulates whole body insulin sensitivity^11^. PGC-1α provides a platform for the recruitment of regulatory protein complexes that exert powerful effects on gene transcription, particularly in regulation of PPARs expression^2, 9^. Also, GLUT-4 and AMPK are cellular energy sensors that regulate glucose and lipid metabolism. The activation of GLUT-4 and AMPK changes many metabolic reactions that would be beneficial to type 2 diabetes and metabolic syndrome, including, increased glucose uptake and fatty oxidation in skeletal muscle^20^. But recent studies show that they decrease expression of PPAR-α, -β/δ, -γ, PGC-1α, GLUT-4, and AMPK in the development of muscular insulin resistance ^20, 27^.

Exercise training also induces several adaptations, such as promoting glucose uptake, insulin sensitivity and insulin signaling pathway in skeletal muscle. In addition, exercise training elevates GLUT-4, AMPK, PPAR-β/α, and PGC-1α activity ^25, 31^.

The potential for exercise training and rosiglitazone to improve skeletal muscle insulin sensitivity by multiple mechanisms raises the possibility that these treatments may also produce additive effects. However, the mechanism by which these therapies increases glucose uptake and improve insulin sensitivity in skeletal muscle is still unclear. Accordingly, the present study was performed to examine the independent and interactive effects of exercise training and rosiglitazone treatment on the expression of PPAR-α, -β/δ, -γ, PGC-1α, GLUT-4 and p-AMPK in skeletal muscle of induced diabetic obese Zucker rats.

## METHODS

### Animals

All experimental procedures performed for this study were approved by the Institutional Animal Care and Use Committee at the Chonbuk National University (2009-1-0074). Male obese Zucker(fa/fa) rats (Samtako, Korea) with body weight of 241.8 ± 7.9 g (mean ± SD) were randomly divided into 5 experimental groups: sedentary untreated controls (n=10), streptozotocin (STZ) treated groups (n=10), STZ plus rosiglitazone treated groups (n=10), STZ plus exercise groups (n=10), and STZ plus exercise plus rosiglitazone treated group (n=10). Diabetes was induced through a single injection of STZ (50 mg/kg, citrate buffer pH7.4) (Sigma, Co) in the tail vein 1. In all animals, ad libitum feeding was resumed 6h after injection. Three days after STZ injection, blood glucose levels were determined with an Accu-Check Compact kit (Roche Diagnostic GmbH, Mannheim, Germany) on blood collected from the tail vein followed an overnight fast. A blood glucose level > 200 mg/μl was considered indicative of diabetes and blood glucose level was measured daily at 09:00~10:00 AM through the duration of experiment^24^. Rats were treated daily for 6weeks by oral gavage with either vehicle, which consisted of 0.5% carboxymethylcellulose (100μl/100g body mass) or 3 mg/kg rosiglitazone (GlaxoSmith Kline, Steneage, U.K) suspended in an equal volume of carboxymethylcellulose ^21^. The trained rats were subjected to 1h of swimming daily, in water at 32±1℃, with attached weight corresponding to 5% of body weight. Exercise training occurred 5days/week for 6 weeks ^23^.

### Skeletal muscle tissues sampling

After 6 weeks of exercise training, rats were killed by i.p. injection of an over-dose of zoletil plus rompun. The extensor EDL and SOL muscles were excised, rinsed in sterile saline, blotted with gauze, and frozen in liquid nitrogen within 15s. The frozen muscle was stored in sterile cryogenic vials containing liquid nitrogen until protein extraction.

### Protein isolation and western blotting

The skeletal muscles were homogenized in a buffer containing 150mM EDTA, 50mM Tri-HCl, 1%-NP 40, 1mM aprotinin, 0.1mM leupeptin, and 1mM pepstatin (pH 8.0). After centrifugation at 14,000g for 30min, the Tripton X-100 soluble fractions (20 μg protein) were electrophoresed in 10% SDS-polyacrylamide gels under non-reducing conditions, in duplicate, comprising a total of eight sets of gels. After overnight electrotransfer, membranes were blocked using 5% skim milk in phosphate-buffered saline (PBS, pH7.4) and incubated with polyclonal PPAR-α (Santacuz, CA, USA, 55kD), PPAR-β/α (Santacuz, CA, USA, 52kD), PPAR-γ (Santacuz, CA, USA, 54kD), PGC-1α (Santacuz, CA, USA, 90kD), GLUT-4 (Santacuz, CA, USA, 50~63kDkD), adiponectin (Cell signaling, Beverly, MA, USA, 30kD), and p-AMPK-α2 antibody (Cell signaling, Beverly, MA, USA, 63kD) at 5 μg/ml dilution in PBS for 1h at room temperature. This was followed by 1 x 15 min and 2 x 5 min washes with PBS plus 0.1% Tween 20. The membrane was then incubated with goat anti-rabbit IgG conjugated with horseradish peroxidase at 1:1,000 dilution in PBS for 1h. Reactive bands were detected by chemiluminescence with Kodak film.

### Statistics

Statistical evaluation of the data was performed by analysis of variance followed by Scheffe’s post hoc test. Data were presented as mean ± SEM, and differences were considered significant at the α=0.05 level of confidence.

## RESULTS

### Effect of exercise training and rosiglitazone on body weight and plasma glucose levels

Body weight decreased after 4weeks in diabetic-rats, as compared with normal rats; whereas, it was significantly increased by rosiglitazone treatment at all periods. The weight significantly decreased in rats subjected to exercise and exercise combined with rosiglitazone than in rats treated with rosiglitazone alone. Diabetic induced-rats by streptozotocin treatment showed significantly increased level of blood glucose. Exercise training and rosiglitazone treatments led to reduced blood glucose level. Particularly, exercise training combined with rosiglitazone treatment group showed reduced blood glucose level, as compared to all other groups except normal group (Table 1).

**Table 1.** Changes of weight and glucose concentration after exercise training (EXER), rosiglitazone (ROSI), and exercise training plus rosiglitazone (ROSI+EXER) in diabetic induced-obese rats by streptozotocine (STZ) treatment.

Groups	Weight (g)			
	0 week	2 week	4 week	6 week
Control	244.35 ± 8.10	288.32 ± 21.76	384.49 ± 18.45	487.36 ± 10.84
STZ+MDSO	243.26 ± 10.01	287.25 ± 22.29	374.51 ± 18.99	475.98 ± 21.53
STZ+ROSI	241.35 ± 8.78	327.88 ± 38.72	419.62 ± 15.54^ab^	559.41 ± 21.93^ab^
STZ+EXER	245.81± 7.51	292.33 ± 17.69	379.26 ± 14.27^c^	456.35 ± 15.79^ac^
STZ+ROSI+EXER	241.73 ± 7.51	292.01 ± 23.47	364.19 ± 25.72^c^	423.62 ± 28.71^abcd^

Groups	Glucose (mg/dl)			
	0 week	2 week	4 week	6 week
Control	113.01 ± 8.27	109.67 ± 8.15	115.14 ± 6.57	132.40 ± 11.35
STZ+MDSO	366.03 ± 18.88^a^	382.28 ± 13.86^a^	409.46 ± 10.97^a^	475.39 ± 20.39^a^
STZ+ROSI	373.53 ± 18.96^a^	363.25 ± 18.18^a^	306.99 ± 12.76^ab^	312.98 ± 14.35^ab^
STZ+EXER	358.88 ± 13.17^a^	350.02 ± 14.35^ab^	336.48 ± 19.48^abc^	292.25 ± 7.15^abc^
STZ+ROSI+EXER	367.71 ± 23.20^a^	344.75 ± 20.33^ab^	311.59 ± 10.82^ab^	271.22 ± 14.48^abcd^

a, b, c, and d: p<0.05 vs. control, STZ+DMSO, STZ+ROSI, STZ+EXER, and STZ+ROSI+EXER respectivly.

### Effect of exercise training and rosiglitazone on of PPAR-α, PPAR-β/δ, PPAR-γ, adiponectin expression in skeletal muscles

**Figure 1.**
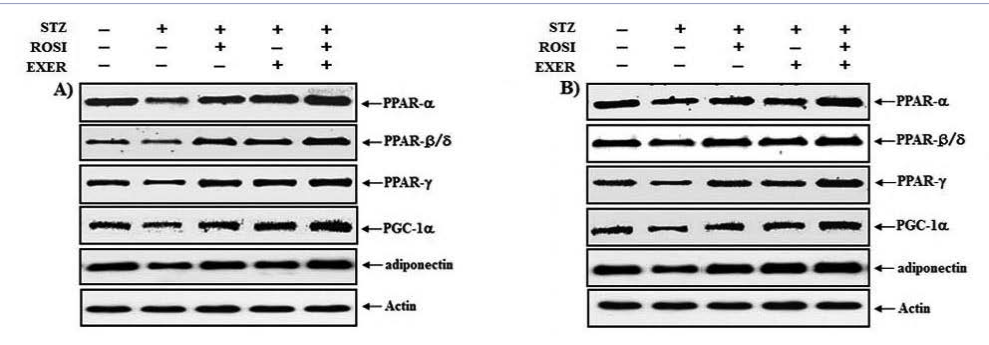
Representative expression pattern of PPAR-α, -β/δ, -γ, PGC-1α and adiponectin in extensor dogitorium longus (A) and soleus (B) muscle after exercise training (EXER), rosiglitazone (ROSI), and exercise training plus rosiglitazone (ROSI + EXER) in diabetic induced-obese rats by streptozotocine (SZT) treatment.

Expression of PPAR-α, -β/δ, -γ, PGC-1αμ, and adiponectin were significantly decreased in EDL and SOL muscle of diabetic induced-rats, as compared with normal rats (Fig. 1, 3, 4, 5). Expression of PPAR-α, -β/δ, -γ, PGC-1αμ, and adiponectin in all muscle were increased in exercise, rosiglitazone, and exercise training combined with rosiglitazone group than in normal and diabetic groups after 6weeks. Especially, exercise training combined with rosiglitazone treatment group showed more elevated expression of PPAR-α, -β/δ, -γ, PGC-1αμ, and adiponectin in EDL and SOL muscle than other groups after 6weeks (Fig. 1, 3, 4, 5, 6, 7).

**Figure 2.**
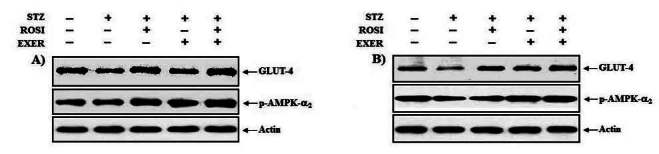
Representative expression pattern of GLUT-4 and pAMPK-α_2_ in extensor dogitorium longus (A) and soleus (B) muscle after exercise training (EXER), rosiglitazone (ROSI), and exercise training plus rosiglitazone (ROSI + EXER) in diabetic induced-obese rats by streptozotocine (SZT) treatment.

**Figure 3.**
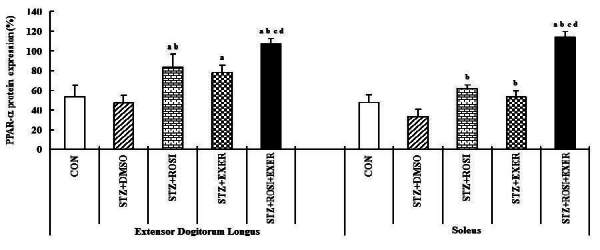
PPAR-α expression in extensor dogitorium longus (A) and soleus (B) muscle after exercise training (EXER), rosiglitazone (ROSI), and exercise training plus rosiglitazone (ROSI + EXER) in diabetic induced-obese rats by streptozotocine (SZT) treatment. a, b, c, and d: p<0.05 vs. control, STZ+DMSO, STZ+ROSI, STZ+EXER, and STZ+ROSI+EXER respectively.

**Figure 4.**
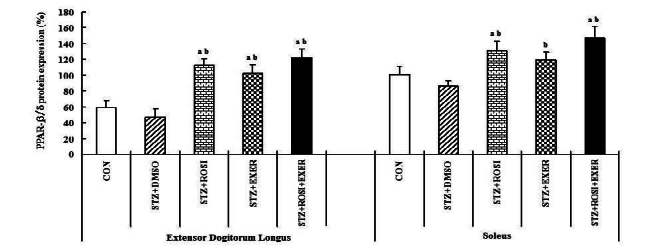
PPAR-β/δ expression in extensor dogitorium longus (A) and soleus (B) muscle after exercise training (EXER), rosiglitazone (ROSI), and exercise training plus rosiglitazone (ROSI + EXER) in diabetic induced-obese rats by streptozotocine (SZT) treatment. a, b, c, and d: p<0.05 vs. control, STZ+DMSO, STZ+ROSI, STZ+EXER, and STZ+ROSI+EXER respectively.

**Figure 5.**
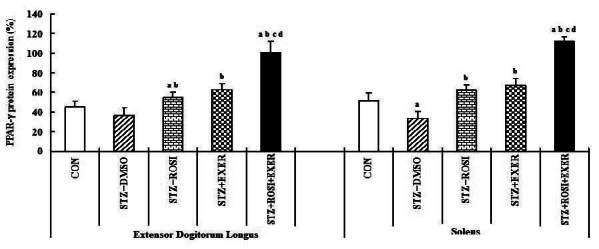
PPAR-γ expression in extensor dogitorium longus (A) and soleus (B) muscle after exercise training (EXER), rosiglitazone (ROSI), and exercise training plus rosiglitazone (ROSI + EXER) in diabetic induced-obese rats by streptozotocine (SZT) treatment. a, b, c, and d: p<0.05 vs. control, STZ+DMSO, STZ+ROSI, STZ+EXER, and STZ+ROSI+EXER respectively.

**Figure 6.**
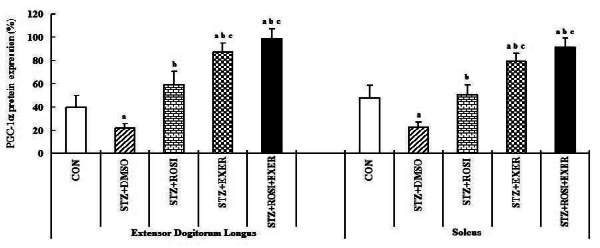
PGC-1α expression in extensor dogitorium longus (A) and soleus (B) muscle after exercise training (EXER), rosiglitazone (ROSI), and exercise training plus rosiglitazone (ROSI + EXER) in diabetic induced-obese rats by streptozotocine (SZT) treatment. a, b, c, and d: p<0.05 vs. control, STZ+DMSO, STZ+ROSI, STZ+EXER, and STZ+ROSI+EXER respectively.

**Figure 7.**
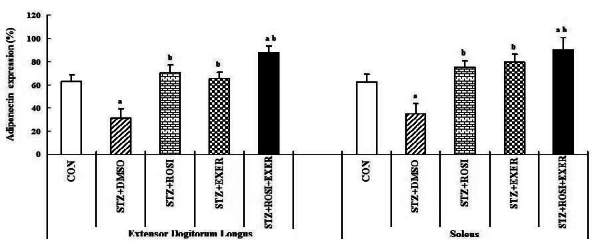
adiponectin expression in extensor dogitorium longus (A) and soleus (B) muscle after exercise training (EXER), rosiglitazone (ROSI), and exercise training plus rosiglitazone (ROSI + EXER) in diabetic induced-obese rats by streptozotocine (SZT) treatment. a, b, c, and d: p<0.05 vs. control, STZ+DMSO, STZ+ROSI, STZ+EXER, and STZ+ROSI+EXER respectively.

### Effect of exercise training and rosiglitazone on GLUT-4 and on p-AMPK-α2 expression in skeletal muscles

**Figure 8.**
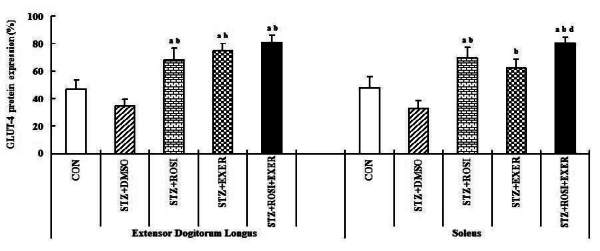
GLUT-4 expression in extensor dogitorium longus (A) and soleus (B) muscle after exercise training (EXER), rosiglitazone (ROSI), and exercise training plus rosiglitazone (ROSI + EXER) in diabetic induced-obese rats by streptozotocine (SZT) treatment. a, b, c, and d: p<0.05 vs. control, STZ+DMSO, STZ+ROSI, STZ+EXER, and STZ+ROSI+EXER respectively.

**Figure 9.**
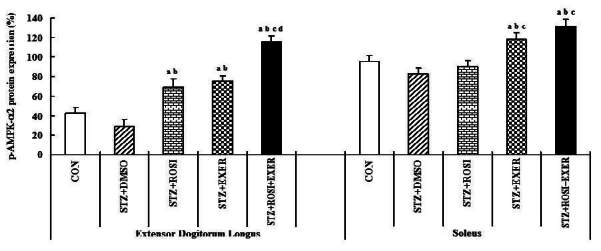
p-AMPKα_2_ expression in extensor dogitorium longus (A) and soleus (B) muscle after exercise training (EXER), rosiglitazone (ROSI), and exercise training plus rosiglitazone (ROSI + EXER) in diabetic induced-obese rats by streptozotocine (SZT) treatment. a, b, c, and d: p<0.05 vs. control, STZ+DMSO, STZ+ROSI, STZ+EXER, and STZ+ROSI+EXER respectively.

Compared with normal rats, muscle expression of GLUT-4 protein was reduced in diabetic induced-rats, as compared with normal rats (Fig. 2, 8); whereas, exercise alone led to an increment; in addition, exercise training combined with rosiglitazone treatment group showed higher GLUT-4 expression than other groups (Fig. 2, 8). Muscle expression of p-AMPK-α2 was decreased in diabetic than in normal rats. In EDL muscle, p-AMPK-α2 expression was significantly increased in exercise, rosiglitazone, and exercise training combined with rosiglitazone treated group, as compared with normal and diabetic induced-rats; whereas, p-AMPK-α2 expression in SOL was significantly elevated in exercise and exercise training combined with rosiglitazone treatment group. Exercise alone and exercise training combined with rosiglitazone treatment groups showed higher p-AMPK-α2 expression in all muscles, as compared to other groups (Fig. 2, 9).

## DISCUSSION

The major findings of the present study are that s streptozotocin-induced diabetic Zucker rats showed decreased body weight. Rosiglitazone treatment led to increased body weight. Plasma glucose level of the induced diabetic Zucker rats increased in all periods. Exercise training with rosiglitazone treatment significantly decreased the body weight and plasma glucose level. Also, exercise training and rosiglitazone treatment showed increased expression of PPAR-α, -β/δ, -γ, PGC-1α, adiponectin, GLUT-4, and p-AMPK-α2 in skeletal muscles, as compared with the induced diabetic Zucker rats. Particularly, combination treatment of exercise training and rosiglitazone showed activated expression of these molecules in skeletal muscles than exercise training and rosiglitazone alone. These results indicated that exercise training combined with rosiglitazone might have additive effects for the therapy of diabetes through the improvement of glucose uptake and insulin sensitivity in skeletal muscle.

Rosiglitazone is known to improve insulin sensitivity through the activation of PPARs^11^. Despite its proven efficacy, widespread use of rosiglitazone for diabetes is limited by their propensity to promote weight gain^10, 32^. In this study, a substantial weight gain was observed in subjects treated with rosiglitazone; however, exercise training combined with rosiglitazone treatment leads to controlled reduction of body weight. Also, plasma glucose levels were higher in induced diabetic Zucker rats than normal rats. Exercise training combined with rosiglitazone treatment resulted in significantly decreased plasma glucose level. These results suggested that combined therapy can minimize the side effects such as weight gain with rosiglitazone treatment and can positively ameliorate the hyperglycemia and enhanced glucose uptake and transport in skeletal muscle.

Obesity and type II diabetes showed excess lipid accumulation, reduced oxidative capacity of mitochondria and impaired glucose transport in skeletal muscle^28^. The improvement of glucose transport and fatty acids oxidation are an important therapeutic goal in obesity and diabetes patients. Exercise training and rosiglitazone are two well-known treatments for improving glucose transport and fatty acids oxidation independently. Therefore, combination of exercise training with rosiglitazone could affect glucose transport and fatty acids oxidation in diabetic patients, which would be of therapeutic importance. To test this idea, we treated diabetic induced-obese Zucker rats with exercise training, rosiglitazone, and exercise training combined with rosiglitazone.

PPARs, PGC-1α and adiponectin are major factors involved in the improvement of insulin sensitivity and fatty acids oxidation in skeletal muscle. The PPAR-α, -β/δ, and PPAR-α isoforms are differentially expressed in tissues that have various functions in lipid metabolism and glucose homeostasis. PGC-1α regulates PPAR-target genes involved in fatty acid oxidation, as well genes involved in glucose transport^17^. Expression of PPAR-α, -β/γ, PPAR-α, and PGC-1α in skeletal muscle was significantly lower in the diabetic state. Reduced PPAR-α, -β/δ, -γ, PGC-1α, and PGC-1α in the diabetes state is followed by increased insulin tolerance and reduction of fatty oxidation in skeletal muscle^25-27, 29^ due to their in glucose transport to skeletal muscle and mitochondrial function of skeletal muscle. In the present study, the muscle expression of PPAR-α, -β/δ, -γ, and PGC-1 was reduced in induced diabetic rats; whereas, exercise training and rosiglitazone treatment increased expression of PPAR-α, -β/δ, -γ, and PGC-1α expression in muscles, as compared with normal and diabetic induced-rats. Increased levels of PPAR-α, -β/δ, -γ, and PGC-1α after exercise training and rosiglitazone treatment may be the result of improved insulin sensitivity and mitochondrial function. Particularly, the additive effects of combination therapy raises the possibility that exercise training with rosiglitazone treatment in diabetes patients could have substantial therapeutic effects to improve glucose transport and mitochondrial function. Also, Hevener et al (2000) reported additive effects of the combination of exercise and rosiglitazone treatment to improve insulin sensitization. We observed that combination therapy significantly increased the expression of PPAR-α, -β/δ, -γ, and PGC-1α expression in muscles. In the present study, expression of PPAR-α, -β/δ, -γ, and PGC-1α after combination therapy, may imply an amelioration of insulin resistance and improvement of mitochondrial function in skeletal muscle of obesity and diabetes.

Adiponectin is an adipose-derived peptide that plays an important role in glucose and lipid homeostasis both directly and as a modulator of insulin sensitivity^35^. Adiponectin levels decrease in parallel with the development of insulin resistance and thus prior to the onset of diabetes^14, 22^. Administration of adiponectin is accompanied by lower plasma glucose levels, as well as increased insulin sensitivity. Many studies have investigated the possible link between adiponectin level or adiponectin receptor (AdipoR) expression and the insulin-sensitizing actions of exercise and TZDs^4, 34^. Bluher et al. (2006) demonstrated that 4weeks training in diabetic patients caused a significant increase in AdipoR and AMPK expression of skeletal muscle, which may mediate the adiponectin-stimulated fatty acids oxidation and glucose uptake. In this study, we showed that adiponectin expression in skeletal muscle was significantly decreased in induced diabetic Zucker rats, as compared with normal rats; whereas, exercise, rosiglitazone, and exercise combined with rosiglitazone treatment showed significant increases in adiponectin expression. These results indicated that exercise training combined with rosiglitazone is positively associated with improvement in glucose uptake and fatty acids oxidation via increased adiponectin expression.

**Figure 10.**
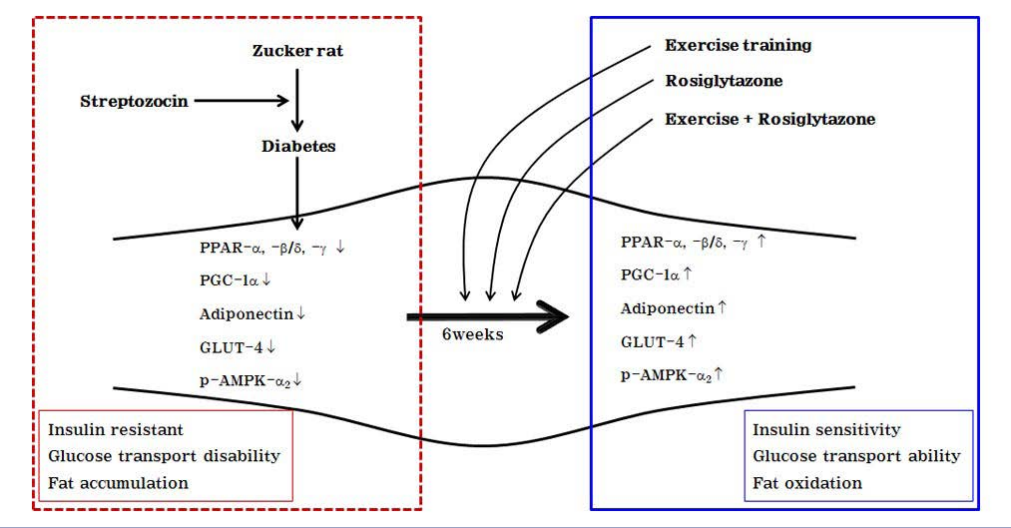
Schematic representation of insulin sensitivity and glucose transport in skeletal muscle of diabetic induced-rat by exercise training, rosiglitazone, and exercise training combined with rosiglitazone during 6weeks.

GLUT-4 is an isoform of glucose family found primarily in insulin sensitive tissues. Insulin-stimulated glucose uptake occurs primarily via the translocation of GLUT-4 to the plasma membrane^3^. Previous studies showed that the decrease in GLUT-4 protein levels in skeletal muscle of diabetic obese rats plays a role in the level of insulin resistance^36^. AMPK is a fuel sensing enzyme that is implicated in insulin sensitivity and lipid homeostasis. AMPK activation causes many metabolic changes including increased glucose uptake and metabolism by increasing GLUT-4 expression and increased oxidation of fatty acids. Therefore, AMPK expression would be beneficial to individuals with type 2 diabetes and metabolic syndrome^12^. A recent study showed that AMPK activation is impaired in skeletal muscle from obese type 2 diabetics^7^. Our results likewise indicated that GLUT-4 and p-AMPK-α2 expression significantly decreases in muscles of induced diabetic rats, as compared with normal rats. These results are consistent with the possibility that a deficiency of GLUT-4 and p-AMPK-α2 expression contributes to decreased glucose transport and increased insulin resistance in diabetic obese rats, because activation of GLUT-4 and AMPK in skeletal muscle may enhance insulin-stimulated glucose transport and glycogen content and lead to increases in fat oxidation, induction of PGC-1α and genes governing mitochondrial biogenesis and enzymes of oxidation phosphorylation^6, 13, 16, 18^. However, treatment with exercise, rosiglitazone, and exercise combined with rosiglitazone augment GLUT-4 and p-AMPK-α2 expression in skeletal muscle of diabetic obese rats.

In summary, exercise training and rosiglitazone treatment activated PPAR-α, -β/δ, -γ, PGC-1α, adiponectin, GLUT-4, and p-AMPK-α2 expression, which was associated with improvement of glucose transport and fatty acids oxidation. Furthermore, the combination of these two therapies led to essential improvement of insulin sensitivity in induced diabetic obese rats. Thus, combination therapy is potentially a key therapeutic strategy of diabetes.
